# Quality of antenatal care and household wealth as determinants of institutional delivery in Pakistan: Results of a cross-sectional household survey

**DOI:** 10.1186/s12978-016-0201-5

**Published:** 2016-07-19

**Authors:** Sohail Agha, Emma Williams

**Affiliations:** The Bill and Melinda Gates Foundation, Seattle, USA; Jhpiego, 1615 Thames St., Baltimore, MD 21231 USA

**Keywords:** Antenatal care, Delivery care, Birth, Quality, Household survey, Pakistan, Asia

## Abstract

**Background:**

Pakistan has a high burden of maternal and newborn mortality, which would be largely preventable through appropriate antenatal and delivery care. While the influence of socio-economic status on institutional delivery is well established in the literature, relatively little is known about the relationship between the quality of antenatal care and institutional delivery.

**Methods:**

A household survey of 4,000 currently married women who had given birth in the two years before the survey was conducted in Sindh province in 2013. The survey collected data on socio-economic and demographic variables, the quality of antenatal care provided during a woman’s last pregnancy and whether she delivered at a health facility. Logistic regression was used to estimate adjusted odds ratios and 95 % confidence intervals around independent variables for institutional delivery.

**Results:**

In the multivariate analysis, a variable measuring quality of antenatal care showed the strongest association with institutional delivery. Moreover, there was a dose-response relationship between the number of elements of quality provided and the odds of institutional delivery: receiving one element of quality increased the odds of institutional delivery 1.7 times, receiving three elements increased the odds 3.8 times and receiving seven elements increased the odds 10.6 times. Household wealth had a statistically significant relationship with institutional delivery but the effect was weaker than that of quality of care. Urban-rural differentials in institutional delivery did not remain significant after adjusting for household wealth and education.

**Conclusions:**

The quality of antenatal care provided to a woman during her pregnancy is more strongly associated with institutional delivery than household wealth. Improving the quality of care at health facilities in Sindh should be the foremost priority. Improving the quality of antenatal care services is likely to contribute to rapid increases in skilled birth attendance and better health outcomes for women and children.

## Plain language summary

Delivering in a health facility can prevent women and newborns from dying during or after childbirth. In Pakistan, 71 % of urban women and 45 % of rural women deliver in a facility. Although most Pakistani women receive antenatal care, the quality of care varies. The purpose of this study was to determine if women who receive higher quality antenatal care are more likely to deliver in a health facility.

We conducted a household survey of 4,000 currently married women who had given birth in the two years before the survey was conducted in Sindh province in 2013. The survey collected data on characteristics of the women and their families, the quality of antenatal care provided during their last pregnancy and whether they delivered at a health facility. Statistical methods were used to determine if women who received higher quality antenatal care were more likely to deliver in a facility, after controlling for other factors that are known to influence place of birth, such as household wealth.

A variable measuring quality of antenatal care showed the strongest association with institutional delivery. Moreover, as the number of elements of quality received increased, the likelihood of institutional delivery also increased. This study concluded that the quality of antenatal care provided to a woman during her pregnancy is more strongly associated with institutional delivery than household wealth. Improving the quality of care at health facilities in Sindh, Pakistan, should be the foremost priority.

## Background

Pakistan has been steadily falling behind its South Asian neighbors in reducing the levels of maternal and neonatal mortality [1]. By contrast with Nepal and Bangladesh which experienced substantial declines in maternal and child mortality after 1990 [2], neonatal mortality increased in Pakistan after 1990 [3] while the decline in maternal mortality has been extremely slow [4]. Studies have identified economic and social barriers [5, 6] and poor quality service provision [7, 8] as factors responsible for Pakistan’s limited progress in improving maternal and child health outcomes.

Institutional delivery is considered a key indicator of progress in reducing maternal mortality [9]. Using data on births from 2002 to 2007, two studies examined correlates of institutional delivery in Pakistan. These studies found significant relationships between household wealth and institutional delivery and between education and institutional delivery. Although both factors are important, the effect of wealth was particularly strong, leading the authors to conclude that many Pakistani women chose to deliver at home because of their inability to pay the high cost of delivery at a health facility [9, 10].

Evaluations of demand side financing interventions have shown that once economic barriers are removed, rapid increases in institutional delivery can occur in Pakistan within a short time frame [11, 12]. These findings are consistent with the finding that a family’s financial status is a primary determinant of the place of delivery. Rural residence and parity also have significant effects on institutional delivery [13, 14]. While the influence of socio-economic status on institutional delivery is well established in the literature, relatively little is known about the relationship between the quality of antenatal care and institutional delivery. This omission is surprising given the importance assigned to quality of care and client satisfaction in models of health service utilization [14, 15].

A recent Tanzanian study, which provided women’s accounts of poor birth care, illustrated the importance of quality of care in determining whether a woman delivers at a health facility. This study found that poor birth experiences undermined the reputation of the health system, lowered community expectations of the efficacy of institutional delivery and contributed to sustained high levels of home delivery. Women who experienced poor quality services had lower trust in health facilities and were pushed towards home delivery [16]. These findings are consistent with findings of a recent evaluation of a well-funded, conditional cash transfer project in Gujrat, India which aimed to increase the institutional delivery rate among the poorest women. The intervention failed to show any effect on the rate of increase of institutional delivery. A principal explanation given for the lack of impact of the intervention was that the quality of care remained poor throughout the period of implementation [15].

The lack of attention to quality as a factor influencing institutional delivery is not just limited to Pakistan but is a shortcoming of many studies that have looked at correlates of institutional delivery. Studies have suggested that a link exists between quality of care and institutional delivery but have not presented empirical evidence to support this hypothesis [17, 18]. A few localized studies with small sample sizes have examined the relationship between quality of antenatal care and institutional delivery. Using a sample of low to middle income women in a city in Uttar Pradesh, India, Bloom et al. developed an index of antenatal care utilization which included both the frequency of antenatal care use and the content of care received by women [19]. Among women who received a high level of antenatal care – those in the 75th percentile of the index - the odds ratio of being assisted by a trained provider was four times as high as that of women who received a low level of care (those in the 25th percentile of the index). A recent study, conducted in three districts of Tanzania which had high levels of institutional delivery rates, examined the association between quality of care and institutional delivery. The authors constructed an index of quality on the basis of services received by women during their antenatal care visits. They found that the institutional delivery rate was dependent on the quality of antenatal care provided to women [20]. Other than a handful of these small-scale studies, however, the relationship between quality of care and institutional delivery remains largely unexplored.

Studies in Pakistan which have examined the correlates of institutional delivery have been based on births which occurred between 2002 and 2007 [9, 10]. There has been a substantial increase in institutional delivery in Pakistan after 2007, from 59 to 71 % in urban areas and from 28 to 45 % in rural areas [3].

This is perhaps the first study in Pakistan that explicitly models the effect of quality of antenatal care on institutional delivery. The study examines the influence of quality care on the choice of place of delivery using a representative household survey of Sindh province conducted in 2013. Sindh province, comprising about 24 % of the population of the country, has mirrored the increase in the institutional delivery rate in Pakistan. While looking at the effects of quality on institutional delivery, our study takes into account the role of a range of other factors which are known to influence a woman’s decision to deliver at a health facility.

## Methods

### Data source and outcome measures

The Maternal and Child Health Program Indicator Survey, a household survey using a shorter version of the Pakistan Demographic and Health Survey (DHS) 2006-07 questionnaire, was conducted in June-July 2013 by MCHIP/Jhpiego as a baseline for a USAID-funded 5 Year MCH Program in Sindh Province. The survey was a provincially representative, multi-stage, cluster sample comprising of interviews with 4,000 currently married women who had a live birth in the two years before survey (for details of sampling methodology and survey implementation see Agha and Williams [21]). The study was approved by the Johns Hopkins University School of Public Health IRB (IRB00005002) and the National Bioethics Committee of Pakistan.

All study participants were married women, aged 15–49, who had had a live birth during the previous two years and lived in a house that was sampled. Because of low female literacy in the study area, interviewers obtained oral consent from each prospective participant prior to conducting an interview, by reading aloud the informed consent script to the prospective participants. Interviewers were instructed to give potential participants a chance to ask questions before providing consent. Interviewers reassured women that they were not obligated to take part in the study and could stop the interview at any time. Interviewers signed a consent form for each participantand kept the form in the study records.

The outcome of interest for this study is institutional delivery. Independent variables included in the analysis are urban/rural residence, woman’s age, number of living children, respondent’s education, household wealth (in quintiles) and whether the respondent received maternal or child health information from an outreach worker. The woman’s age was categorized as 15-24, 25-34, or 35 years and older. Level of education has the highest level completed and was categorized as none, primary or middle school, or secondary and higher. Household wealth quintiles were calculated using principal component analysis, using the same methodology of as the Demographic Health Surveys [3]. Information from an outreach worker could have been received any time during the year preceding the survey and could have included counseling related to a facility birth or other topics.

Quality of care is defined as the receipt of recommended services during antenatal care; one recent study has used a similar approach [22]. This is based on Donabedian’s definition of quality being the extent to which actual care provided is consistent with standards of care [23]. The variable is operationalized as the number of elements of quality of care received by the respondent during antenatal care. The variable is a sum of seven elements of quality that a woman should receive during her pregnancy: urine sample, blood sample, blood pressure, iron tablets, two tetanus shots, measurement of weight and advice about danger signs of pregnancy. These elements of antenatal care service provision are measured by the DHS and were measured in the Maternal and Child Health Program Indicator Survey.

### Data analysis

Bivariate and multivariate logistic regression analysis was conducted with Stata Version 12 (College Station, Texas), using the SVY suite of commands which takes the multistage cluster design of the survey into account when calculating standard errors.

## Results

Column 1 of Table 1 shows the distribution of sample characteristics. About 51 % of married women 15-49 in the sample live in rural Sindh. The distribution of women’s current age was 32 % age 15 to 24, 55 % age 25 to 34, and 13 % age 35 to 49. In our sample, 52 % of women had three or more children.

**Table 1:** Sample distribution, percentage of women who had a facility delivery, and unadjusted odds of facility delivery

	Sample Distribution (*n* = 4,000) (1)	% Who Delivered in a Health Facility (2)	Unadjusted Odds of Facility Delivery (3)	Number of Cases
Residence				
Urban	48.8 %	79.9 %	1.00	1,951
Rural	51.2 %	53.5 %	0.29^***^	2,049
Age				
15-24	32.2 %	70.7 %	1.00	1288
25-34	55.2 %	65.4 %	0.79^**^	2207
35-49	12.6 %	59.9 %	0.62^***^	505
Number of living children				
1	24.5 %	76.5 %	1.00	981
2	23.9 %	71.5 %	0.77^*^	956
3 or more	51.6 %	59.2 %	0.45^***^	2063
Education				
No education	56.6 %	52.1 %	1.00	2,265
Primary or Middle	19.5 %	76.1 %	2.93^***^	779
Secondary or Higher	23.9 %	92.4 %	11.18^***^	956
Wealth quintiles				
First/poorest	20.0 %	40.1 %	1.00	802
Second	20.0 %	52.9 %	1.68^***^	800
Middle	20.0 %	68.0 %	3.18^***^	800
Fourth	19.9 %	78.8 %	5.57^***^	798
Fifth/richest	20.0 %	92.4 %	18.30^***^	800
Received MCH information from an LHW				
No	86.6 %	65.4 %	1.00	3,465
Yes	13.4 %	73.3 %	1.45^**^	535
# of elements of quality of care received				
Did not receive any element of care	13.8 %	26.1 %	1.00	551
One	10.9 %	38.6 %	1.78^***^	437
Two	7.6 %	49.2 %	2.73^***^	303
Three	7.9 %	63.1 %	4.81^***^	314
Four	10.4 %	68.0 %	6.02^***^	415
Five	17.4 %	81.9 %	12.79^***^	696
Six	22.9 %	88.6 %	21.84^***^	918
Seven	9.1 %	91.0 %	28.62^***^	366
Total	100.0 %	66.4 %		4,000

^*^ *p* < 0.05, ^**^ *p* < 0.01, ^***^ *p* < 0.001

About 57 % of women in the sample had no formal education. About 13 % of women had received information on maternal and child health (MCH) from a Lady Health Worker (LHW) – a community health worker employed by the government and tasked with extending services to women in low income urban and rural areas - in the last 12 months.

The quality of care variable measures the number of elements of quality a woman received during her last pregnancy. About 14 % of all women in the sample did not receive any of these seven services, 19 % received one or two services, 18 % received three or four services, 17 % received five services, 23 % received six services, and 9 % received seven services. Only about 2 % of women received at antenatal care home at home, while the rest were at various types of health facilities (data not shown).

Table 1 shows the percentage of women who delivered at a health facility by socio-demographic characteristics and quality of care. The table also shows cross tabulations between independent variables and institutional delivery and the unadjusted odds of institutional delivery. About 54 % of rural women delivered at a health facility compared with 80 % of urban women (odds ratio = 0.29, *p* < 0.001). Women at older ages were less likely to deliver at a health facility: about 60 % of women aged 25-34 delivered at a facility, compared to 71 % of women ages 15-24 (odds ratio = 0.62, *p* < 0.001). Women at higher parities were less likely to deliver at a health facility: 59 % of women with three or more children delivered at a health facility, compared to 77 % of women who had their first birth (odds ratio = 0.45, *p* < 0.001). Among all women who did not deliver at home, 75 % delivered at a private hospital or clinic, 23 % delivered at a government hospital, and the remainder delivered in other types of health facilities (data not shown).

Bivariate analysis shows that institutional delivery increases with education: 92 % of women with secondary or higher education being delivered in a health facility compared to 52 % of women with no formal education (odds ratio = 11.18, *p* < 0.001). Wealth had a powerful association with institutional delivery, with 92 % of all women living in households in the fifth/richest quintile delivering at a health facility, compared to 40 % of women in the first/poorest quintile (odds ratio = 18.28, *p* < 0.001).

Bivariate analysis also shows that quality of care has a powerful relationship institutional delivery: 91 % of women who received seven elements of quality delivered at a health facility compared to 26 % of women who did not receive any element of quality during their pregnancy (odds ratio = 28.62, *p* < 0.001). On average, at the bivariate level, every additional element of quality of care added about 10 percentage points to the rate of institutional delivery.

Table 2 shows the adjusted odds of institutional delivery in Sindh province. After adjusting for education and household wealth, urban women are no different from rural women in their propensity to deliver at a health facility. In other words, the effect of urban residence appears to operate primarily through the education and wealth of women who live in urban areas. The effect of age changes direction after adjusting for socio-demographic variables in the model, with women 35 to 49 being more likely to deliver at a health facility after adjusting for education and wealth. This suggests that were it not for their being less educated and for their being poorer, women 35-49 would have been more likely to deliver at a health facility.

**Table 2:** Adjusted odds of facility delivery, by socio-demographic factors and quality of care received during pregnancy

	Adjusted Odds Ratios	95 % Confidence Intervals	Number of Cases
Residence			
Urban	1.00		1,951
Rural	1.19	0.87 – 1.61	2,049
Age			
15-24	1.00		506
25-34	1.02	0.83 – 1.26	446
35-49	1.42^*^	1.02 – 1.97	2,056
Number of living children			
1	1.00		981
2	0.76^*^	0.59 – 0.98	956
3 or more	0.56^***^	0.44 – 0.72	2063
Education			
No education	1.00		2,265
Primary or Middle	1.35^*^	1.05 – 1.75	779
Secondary or Higher	2.74^***^	1.95 – 3.87	956
Wealth quintiles			
First/poorest	1.00		802
Second	1.36^*^	1.02 – 1.80	800
Middle	1.63^**^	1.13 – 2.36	800
Fourth	1.95^**^	1.30 – 2.93	798
Fifth/richest	3.78^***^	2.29 – 6.25	800
Received any MCH information from an LHW			
No	1.00		3,465
Yes	1.27	0.95 – 1.70	535
Number of elements of quality of care received			
Did not receive any element of care	1.00		551
One	1.71^**^	1.25 – 2.34	437
Two	2.39^***^	1.70 – 3.35	303
Three	3.92^***^	2.70 – 5.69	314
Four	4.17^***^	2.94 – 5.92	415
Five	7.41^***^	5.35 – 10.26	696
Six	9.71^***^	6.74 – 14.01	918
Seven	10.94^***^	6.40 – 18.69	366
Pseudo R^2^	23.72 %		4,000

^*^ *p* < 0.05, ^**^ *p* < 0.01, ^***^ *p* < 0.001

Parity retains a significant association with institutional delivery: after adjusting for other variables, women with two or more children are less likely to deliver at a health facility than women who have their first birth (odds ratio = 0.62, *p* < 0.001). The effect of education remains significant: women with secondary or higher education have a three times higher odds of institutional delivery compared to women with no formal education. Similarly, the effect of household wealth remains strong and statistically significant: women in the richest quintile have a four times higher odds of institutional delivery compared to women in the poorest quintile.

Even after taking education and household wealth - two important determinants of institutional delivery – into account, quality of care remains strongly associated with institutional delivery. Women who receive one element of quality during an ANC visit have a 1.70 times higher odds ratio of delivering in a health facility (*p* < 0.01), those who receive three elements have a 3.83 times higher odds ratio (*p* < 0.001), those who receive five elements of quality have a 7.32 times higher odds ratio (*p* < 0.001) and those who receive seven elements of quality have an 10.64 times higher odds of delivering in a facility (*p* < 0.001). Overall, the model explains 24 % the variance in the outcome.

## Discussion

Although models of health service access highlight the importance of quality and client satisfaction, quality of care has received scant attention in quantitative analysis of the determinants of institutional delivery. As a result, with the exception of a few empirical investigations based on small samples [19, 20, 22], there is little evidence to illustrate the type of relationship that exists between quality of care and subsequent choice of place of delivery. In Pakistan, health interventions have traditionally had a supply side focus and efforts have not been made to empirically determine whether quality of care influences client satisfaction and subsequent service utilization. The findings of this study, based on a representative sample of women in Sindh province suggest that quality of antenatal care is a powerful determinant of institutional delivery in Pakistan. Indeed, our findings suggest that quality of care has a stronger effect on institutional delivery than either wealth or education. We speculate that quality of care could influence subsequent care-seeking by influencing women’s overall perception of the value of facility-based care and perhaps their confidence in facility-based providers. Greater quality of care, as measured through specific services and counseling about danger signs, also could have been accompanied by better communication with health care providers about the importance of delivery in a health facility. It is possible that, for some women, other factors underlie both receiving higher quality antenatal care and facility delivery; for example, women experiencing complications might receive more screening tests and be more likely to deliver in a facility.

Recent analyses of data from Sindh have shown that the quality of antenatal care is very poor: only 10 % of women who receive antenatal care are provided essential elements of antenatal care during their examinations [21]. Thus, while nearly eight out of 10 women in Sindh receive antenatal care from a skilled provider, only 10 % of these women receive care as per WHO established standards of care.

The lack of documented evidence of successful quality improvement efforts in Pakistan is a matter of enormous concern, as it is globally [1]. There remains an urgent need for programs to demonstrate how quality of maternal and child health services can be improved in Pakistan. It has been suggested that the slow pace of improvement of quality of care has arrested what might otherwise have been a faster decline in maternal mortality [1]. In India, for example, conditional cash transfers to incentivize facility delivery have led to increased coverage, but quality of care remains a concern [24, 25].

The finding that quality of care has a more powerful effect on institutional delivery than either education or household wealth highlights the importance of both intensifying efforts to improve quality and developing systems to measure quality of care. At present, the government’s health information system does not measure quality of care in the public sector while there is no systematic recordkeeping in most private sector facilities. Unless quality is measured, quality improvement targets cannot be set. Nor can quality improvement efforts be resourced adequately unless the extent of the problem is understood. Not placing quality of care front and center in Pakistan’s efforts to reduce maternal and child mortality is reducing the impact of current efforts to improve maternal and child health outcomes in Pakistan.

An important finding of this study is the absence of differentials in institutional delivery between urban and rural Sindh once wealth and education are taken into account. There has been an increase in the institutional delivery rate in Pakistan after 2007 from 34 to 48 % [3]. This trend has been particular strong in rural Sindh, where the institutional delivery rate was 58% in the 2012 Demographic Health Survey and 66 in the 2013 survey reported here [21]. This is perhaps the first study that has examined urban rural differentials in institutional delivery in Sindh using data on births after 2007. That the urban-rural differential in facility delivery disappears after controlling for household wealth and women’s education suggests that women living in urban and rural areas of Sindh were equally likely to deliver at a health facility in 2013 as long as they had the financial means to do so and were aware of the importance of institutional delivery.

### Strengths and limitations

A strength of this study is the use of a large, representative, sample of women from the second largest province of Pakistan, Sindh, which has a population of 45 million. The few studies that have examined the relationship between quality and institutional delivery have been based on small samples from select populations. Another strength of the study is the range of variables used and the use of multivariate analysis to adjust for confounding influences.

Limitations of the study include the cross sectional design of the study and our inability to make causal inferences from the findings. Another limitation of the study is that the quality of care variable is based on retrospective self-report of antenatal care services, which may not capture the actual experience of women who receive those services. Our measure of quality of care included only six aspects services received and one measure of counseling. Hulton has identified various other quality domains not included in this study, such as human and physical resources of the health system, referral systems, information systems, appropriate technologies, management of emergencies, experience of care, cognition, respect, dignity, equity, and emotional support [26]. Similarly, Tuncalp has noted that quality includes both provision of care and experience of care, with several subdomains for each other these, plus competent and motivated human resources and emotional and physical resources [27]. Therefore, the measure of quality presented here is incomplete. However, data on patient experiences are often not collected in large scale surveys because of lack of validated instruments that can be included in survey questionnaires [22].

Women with health complications are more likely to seek both antenatal and delivery care, and this analysis did not control for this underlying factor. It is also known that the “three delays” impact women’s ability to utilize maternity care - we were unable to measure these in the context of this survey [17]. While this study measured quality of care only through clients’ retrospective self-reports, measuring quality of care through direct clinical observation is also an important methodology that has rarely been used in Pakistan; the research team that conducted this study is in the process of publishing findings from a direct clinical observation study in Sindh province.

## Conclusions

The findings of this study show that the effects of quality of care are strong and powerful, and independent of the effects of education, wealth and other variables. Moreover, quality of care appears to have a dose-response relationship with institutional delivery: the better the quality of care provided, the higher the institutional delivery rate. The findings emphasize the importance of having a strong focus on improving quality of antenatal care services in Pakistan in order to drive rapid increases in the institutional delivery rate and improve health outcomes for women and children.
